# How Do Spelling, Handwriting Speed, and Handwriting Quality Develop During Primary School? Cross-Classified Growth Curve Analysis of Children's Writing Development

**DOI:** 10.3389/fpsyg.2021.685681

**Published:** 2021-07-23

**Authors:** Claire Gosse, Michael Parmentier, Marie Van Reybroeck

**Affiliations:** Psychological Sciences Research Institute (IPSY), Catholic University of Louvain, Louvain-la-Neuve, Belgium

**Keywords:** writing development, spelling, handwriting speed, handwriting legibility, longitudinal cross-classified Bayesian structural equation modeling, transcription skills, handwriting quality

## Abstract

**Aim:** Longitudinal studies are rare in the field of writing research, and little is known about the concurrent development of the two transcription skills: spelling and handwriting. This study was designed to provide a comprehensive picture of the development and the longitudinal relations between spelling, handwriting speed, and handwriting quality at the word level.

**Method:** Over a period of 3 years (coh1: Grades 2–4; coh2: Grades 3–5), 117 French-speaking children were assessed on a single-word dictation task. At each testing time, measures of spelling accuracy, handwriting speed, and handwriting quality were collected on 40 words. Words varied in both orthographic and graphic complexity, making it possible to investigate the influence of these levels of complexity on transcription abilities.

**Results:** Linear growth analyses using cross-classified Bayesian structural equation modeling (CC-BSEM) revealed that spelling and speed continued to improve until Grade 5, while handwriting quality reached an early plateau in Grade 2. In the younger cohort, graphic complexity had a significant influence on the pace of development of handwriting speed and on spelling and handwriting quality performance in Grade 2. In the older cohort, a positive relation between spelling and speed and a negative relation between handwriting speed and handwriting quality were found, indicating that fast handwriting is associated with high spelling ability and that fast handwriting is detrimental to handwriting quality. By providing a better understanding of writing development, this study yields innovative findings not only regarding the development of transcription skills but also regarding how spelling, handwriting speed, and handwriting quality can influence each other's performance throughout primary school.

## Introduction

At the beginning of formal education, learning to write occupies a major place in the classroom. Writing is a complex ability that takes time to develop, involving a wide range of cognitive, psychomotor, and perceptual-motor processes (Van Galen's, 1991; Feder and Majnemer, 2007). Learning to write represents a long-lasting challenge for children, which starts with the acquisition of the foundations of writing: spelling and handwriting, the two transcription skills. These transcription skills originate from different domains, with spelling being a language-based component and handwriting a motor-based ability. Van Galen (1991) reference theory of writing represented orthographic and graphomotor skills as separate processes, occurring one after the other in a discrete manner. Since then, studies have demonstrated that spelling and graphomotor processes have influenced each other during the course of writing, challenging this vision of independent processes (Kandel and Perret, 2015; Palmis et al., 2019; Gosse and Van Reybroeck, 2020). However, little is known about the concurrent development of transcription skills, and our understanding of how spelling and handwriting influence each other is limited (Caravolas et al., 2020). Even though the current literature contains studies focused on either children's spelling or handwriting, collecting data that consider both transcription skills together is necessary to understand their relationship. Moreover, although it is a major aspect of handwriting, legibility has only rarely been investigated in typically developing children (Gosse et al., 2018; Caravolas et al., 2020). Indeed, the great majority of studies on handwriting reported results based on the dynamic parameters of handwriting (i.e., speed, pausing). Finally, longitudinal designs are very rare in the field of writing development (Abbott et al., 2010; Bosga-Stork et al., 2016; Barnett et al., 2019), which constitutes a gap in the current literature.

The present study is a longitudinal study aimed at providing a comprehensive picture of the concurrent development of transcription skills at the word level. For the first time, spelling accuracy, handwriting speed, and handwriting quality were studied simultaneously in a single word writing context. By taking into consideration both transcription skills, this study was designed to address the question of the development of each skill and how they relate to each other at different ages. To this end, over a period of 3 years, 117 French-speaking children with no learning difficulties were assessed on a single-word dictation task. At each testing time, measures of spelling accuracy, handwriting speed, and handwriting quality were collected for each word. The words used in the dictation task varied in orthographic and graphic levels of complexity in order to assess their influence on transcription abilities. Cross-classified Bayesian structural equation modeling (CC-BSEM) was used to investigate children's concurrent development of spelling and handwriting, allowing the integration of both the psycholinguistic (i.e., orthographic and graphic levels of complexity at the word level) and longitudinal approaches.

### The Transcription Skills

#### Spelling Accuracy

In all languages, becoming an accurate speller takes several years (Treiman, 2017). At the word-level, children begin spelling acquisition by relying on phonological processes, allowing to build the words by applying phoneme to grapheme correspondences. However, correctly applying these correspondences is not sufficient to master word spelling, as some words have irregular spelling, i.e., a low degree of consistency between phonemes and graphemes (Treiman, 2017). This is especially true in languages like English and French, often referred as opaque orthographic systems (Salas and Caravolas, 2019).

In French, the language of the present study, over half of the words contain phonemes that can be spelled in different ways (Ziegler et al., 1996). For example, there are several phonologically correct ways of spelling the phoneme [ε], using the graphemes *in, ain*, and *ein* like in *vin* (wine), *main* (hand), and *plein* (full). French also contains words with inaudible letters (Sénéchal, 2000; Casalis et al., 2011), like the final silent letters in *lait* (milk) and *bois* (wood). Research conducted on the morphological structure of words have revealed that children use information about morphemes to choose among several possibilities of plausible spellings (Deacon and Bryant, 2006). For example, in the case of the nouns *bois* (wood) and *milk* (lait), clues regarding the silent letters *s* and *t* can be provided using the derived adjectives *boisé* (wooded) or *laiteux* (milky). Children can also use contextual information that they learn implicitly to choose between several plausible spellings, as they are sensitive from a young age to the frequency of orthographic patterns (Pacton et al., 2001; Hayes, 2006). In other words, children can take spelling decisions by selecting the most frequent orthographic patterns among several plausible spellings [e.g., in French, most of the words starting with the sound [ap] are spelled with a double consonant *pp* like in *apprendre* (learn), *appeler* (call), *apparaître* (appear)].

Regarding the pace of spelling development, longitudinal data collection are rare, and results vary depending on the language at stake, as the rate of spelling development depends on the level of orthographic consistency (Salas and Caravolas, 2019). A longitudinal study conducted in French from Grade 1 to Grade 4 revealed that irregular words were still being produced significantly less accurately than regular words at the end of Grade 4 (Sprenger-Charolles et al., 2003). These longitudinal data indicated that the development of spelling was still ongoing at the end of Grade 4. The importance of the regularity parameter for spelling development in French has been highlighted in other psycholinguistic experiments (Tainturier and Rapp, 2001; Martinet et al., 2004). For regular words, i.e., those with a high degree of consistency, spelling is easier and therefore more accurate than it is for irregular words, and this effect is long lasting. Throughout the literacy experience and word-writing practice, children progressively access words' spelling forms quicker, as they can use their orthographic lexicon in long-term memory (Sprenger-Charolles et al., 1998).

#### Handwriting Ability

Handwriting is a psychomotor ability defined by two outcomes: speed and legibility, also referred as handwriting quality (Graham et al., 2006). The handwriting style used by children throughout the world depends on their national education context. In alphabetic contexts, we find two main handwriting styles: cursive vs. script (e.g., respectively, handwriting vs. handwriting). Cursive handwriting implies continuous graphomotor movement, while script handwriting, typical of English-speaking countries, requires pausing between each letter. Moreover, the visual characteristics of letters vary (Morin et al., 2012; Bara and Morin, 2013), and the number of strokes composing each letter can also differ depending on the handwriting style (e.g., cursive *j* has more strokes than script *j*).

Handwriting speed has been the center of many recent experiments in both cursive (e.g., Alamargot et al., 2020; Blampain et al., 2021) and script styles (e.g., Sumner et al., 2014), with digital tablets providing data recordings of the dynamics of handwriting (e.g., pen pressure, speed, writing duration). In contrast, there is a lack of objective and sensitive measures of legibility (Barnett et al., 2018), which presumably explains why it has been overlooked compared to speed. However, it is essential to take an interest in legibility since poor handwriting can have far-reaching consequences for children's self-esteem and academic achievement (Feder and Majnemer, 2007; Medwell and Wray, 2007). The few studies focusing on legibility used criteria that are commonly related to letter formation and to spatial organization within and between words (e.g., unusual letter shapes, size fluctuation, bad letter alignment, abnormal space between letters; Graham et al., 2006; Caravolas et al., 2020). Such criteria allow identifying children who have graphomotor difficulties, leading to illegible handwriting like in the case of developmental coordination disorder or, to a lesser extent, dyslexia (Prunty and Barnett, 2017; Di Brina et al., 2018; Downing and Caravolas, 2020). However, handwriting has never been investigated with such precise legibility criteria in typically developing children, to measure the quality of their handwriting.

Regarding handwriting developmental paths, few studies have examined the growth in handwriting speed and legibility. To the best of our knowledge, only one experiment by Karlsdottir and Stefansson's (2002) has collected longitudinal data covering several school years (Grade 1–5). Handwriting speed and legibility were assessed in over 400 Norwegian children. The results revealed that children's handwriting quality increased rapidly during first grade, with children reaching a plateau at the end of Grade 1. In contrast, the speed of handwriting had a continuous and linear developmental pattern. These results confirmed in part a large cross-sectional experiment conducted by Graham et al. (1998) covering Grades 1–9. Their findings demonstrated a plateau in legibility occurring in the middle grades, not as early as in Karlsdottir and Stefansson (2002) study, but continuing to improve until the later grades. Moreover, Graham et al. (1998) reported that correlations between legibility and speed were weak, which supported the idea that the two components of handwriting have different developmental patterns.

While the psycholinguistic characteristics that determine orthographic complexity are documented (i.e., word regularity), little is known about the parameters that influence the graphic complexity of words. Recent findings have demonstrated that words vary in levels of graphic complexity, depending on the fine graphomotor skills, and motor control abilities implied by the pen stroke trajectory. Specifically, tracing pieces that contain abrupt changes in the pen stroke trajectory (e.g., the angle in *r*) is more difficult than tracing curvy segments (e.g., *c*; Gosse et al., 2018). To the best of our knowledge, no study has ever investigated the development of handwriting by taking into account the different levels of graphic complexity required by handwriting.

#### Spelling and Handwriting Instruction in the Current Context

Handwriting and spelling instruction vary greatly from one country to another, depending on the handwriting style taught and the language at stake. The current study was conducted in a French-speaking context in Belgium. With regard to handwriting instruction, block letters are taught before entering primary school, at the end of Kindergarten. Once in primary school, children all use cursive style exclusively, as script style is never taught in French-speaking Belgian schools. Handwriting instruction occurs during first grade, and according to the national school curriculum, children by the end of Grade 2 are all expected to produce legible handwriting and to master organization and neatness of their sheets of paper. In contrast, spelling instruction in French-speaking Belgium remains the focus of teachers throughout primary school. Typically, children start in Grade 1 by learning the correspondences between phonemes and graphemes, consisting in single letter writing followed by syllable writing. Later, teachers use lists of words that contain similar spelling patterns and progressively increase difficulty, going from highly regular words to irregular words. Spelling instruction also involves teaching contextual spelling rules and morphological principles.

### Relationship Between the Two Transcription Skills

The model of writing by Van Galen's (1991) represents spelling and handwriting as independent processes occurring one after the other. However, the literature contains evidence of interactions between spelling and handwriting processes during the course of writing (Caravolas et al., 2020). Current knowledge regarding the interactions between spelling and handwriting arises from studies that can be classified into two groups. The first group of experiments contained studies related to the capacity theory of writing (McCutchen's, 1996) framework, which contributed to highlighting the crucial role of handwriting automatisation in the development of higher-level writing processes. The second group is composed of psycholinguistic experiments, in which the manipulation of words' characteristics and complexity (either orthographic or graphic) was used to shed light on their impact on spelling and handwriting performances.

#### The Limited Capacity Theory of Writing

According to McCutchen (1996) capacity theory of writing, the lower processes of writing must be automatised to allow higher-level processes to develop. At the beginning of writing instruction, the movements necessary for letter formation are under voluntary control, requiring a large proportion of children's cognitive resources (Jones and Christensen, 1999). Throughout their experience, children progressively automate their handwriting movements, which lightens cognitive constraints and frees up resources for other processes (Graham et al., 1998; Chartrel and Vinter, 2004; Overvelde and Hulstijn, 2011). The positive key role played by handwriting automatisation has been demonstrated for various processes of writing either at the text level on composition quality (Medwell and Wray, 2007; Alves and Limpo, 2015) or at the word level on spelling accuracy (Abbott et al., 2010; Pontart et al., 2013). However, there is no consensus about the age at which this automatisation of handwriting occurs, with data suggesting it would already occur at the end of Grade 1 (Overvelde and Hulstijn, 2011) or after Grade 7 (Alves and Limpo, 2015). As already mentioned above, the way handwriting is taught varies greatly across countries (i.e., script or cursive only or both). This variability may be one plausible explanation of the difficulty of reaching a consensus regarding the age at which handwriting is automatic.

#### Psycholinguistic Approach in Writing Research

Researchers who have adopted a psycholinguistic approach have manipulated the attributes of the words composing the experimental writing tasks (e.g., copying regular vs. irregular words in Kandel and Perret, 2015) to assess their impact on writing performance. Such experiments have led to highly informative findings regarding the relationship between the two transcription skills. Studies conducted in French have revealed that words' orthographic characteristics, especially regularity, influenced handwriting fluency in university students (Delattre et al., 2006) and in children from 8 to 10 years old (Kandel and Perret, 2015). The impact of orthographic complexity on handwriting execution has been demonstrated in adults by neuroimaging evidence (Palmis et al., 2019). The opposite direction of the relationship has also been investigated, revealing that the graphomotor demand was negatively related to grammatical spelling performance in French (Van Reybroeck and Hupet, 2009). Recently, a study conducted in children with and without dyslexia reported evidence of the negative impact of word graphic complexity on spelling accuracy in a sample of typically developing children (Gosse and Van Reybroeck, 2020). More precisely, the authors revealed that words that contained graphically complex segments (i.e., abrupt changes of pen stroke trajectory induced by the presence of letters containing angles like the letters r and b), based on the index of graphic complexity developed in Gosse et al. (2018), were produced with more spelling errors than graphically simple words (i.e., words that are mostly composed of curvy segments like the letters e and c). Taken together, these findings have demonstrated that the orthographic and graphic sides of writing influence each other. However, no experiment has ever measured the influence of both orthographic and graphic features of words within the same writing task.

Finally, a developmental approach can be found in the experiment by Bosga-Stork et al. (2016) conducted with 30 Dutch children. Besides demonstrating children' handwriting, spelling, and motor skill increasing development from Grade 1 to Grade 3, their findings demonstrated that handwriting speed was positively related to spelling accuracy in Grades 1 and 2 and that this correlation was no longer significant in Grade 3. Despite providing longitudinal evidence for the development of handwriting and spelling, their analysis of the relations between the two components were cross-sectional in nature. Their analysis thus failed to properly assess how handwriting and spelling co-develop and their relationships over time. For this reason and because of the small sample size (*N* = 30), this study was qualified by the authors themselves as exploratory. Moreover, this experiment did not take into consideration the quality component of handwriting.

To conclude, while the spelling side of writing is a well-documented ability, little is known about the development of handwriting. Even if spelling and handwriting abilities originate from separate cognitive processes, the studies presented in the above introduction section provided evidence of their interaction during the course of writing. Indeed, psycholinguistic studies revealed a significant influence of the orthographic characteristics of words on handwriting outcomes (e.g., Kandel and Perret, 2015). However, these psycholinguistic studies did not address the issue of the development of spelling and handwriting. The one experiment that used a developmental approach to investigate the concurrent development of spelling and handwriting were of a correlational nature (Bosga-Stork et al., 2016). Moreover, the assessment of handwriting was limited to measures of speed, while handwriting quality has never been investigated in relation to spelling. To the best of our knowledge, the concurrent development of spelling, handwriting speed, and quality has never been investigated with a longitudinal approach. Such data would be a meaningful contribution to the field of research by providing for the first time a comprehensive picture of word writing development.

## The Present Study

The purpose of this study was to better understand the typical development at the word-level of spelling, handwriting speed, and handwriting quality throughout primary school. The current study involved longitudinal data collected from 117 French-speaking children, who used cursive handwriting style only. Standardized control measures of general cognitive and literacy abilities were collected to ensure that all children were typically developing. The experimental task was a single word dictation task that children performed once a year for a period of three consecutive years. Children's spelling accuracy, handwriting speed, and handwriting quality were assessed at each measurement time. Through the manipulation of both orthographic and graphic levels of complexity of words, this study investigates how the orthographic and graphic sides of writing influence the development of the transcription abilities. Spelling and handwriting (both quality and speed) have never been considered in relation to the orthographic and graphic features of words within the same experiment, even more so using longitudinal data collection. The impact of word levels of orthographic and graphic complexity on spelling and handwriting abilities was assessed. Cross-classified Bayesian structural equation modeling was used in the present study to account for (1) the cross-nested structure of the data within children and words at the same time and (2) the repeated-measures longitudinal design and (3) the concurrent development of spelling, handwriting speed and quality. While mixed effects models have been widely used to study cross-classified data structures (Baayen et al., 2008; Judd et al., 2012), the CC-BSEM approach allows greater flexibility in adopting a multivariate structural equation modeling framework (Wickham et al., 2021). To the best of our knowledge, the present study is the first to adopt a CC-BSEM approach to address the question of the developing relationship between spelling, handwriting speed, and handwriting quality. One of the main advantages of our approach is that it allowed us to investigate, at the word level, the typical development of spelling, handwriting speed and quality while, simultaneously, examine how their development is intertwined over time.

Participants were from two cohorts with a 1-year difference (coh1: Grade 2 to Grade 4; coh2: Grade 3–5), implying different degrees of handwriting automatisation. Indeed, coh1 children were in Grade 2 at the start of the study, having received only a year and a half of handwriting instruction, while coh2 children were in Grade 3, having received an additional full year of handwriting experience. Therefore, the level of graphomotor automatisation between the two cohorts was different, being lower in coh1 than in coh2.

The present study addressed the three following research questions:

(i) How do spelling, handwriting speed, and handwriting quality develop at the word level?

Since the present study was conducted in French, an opaque orthography, we expected spelling accuracy to continue growing (Sprenger-Charolles et al., 2003) until the end of the longitudinal study for both cohorts. In line with past research, the same prediction applies for handwriting speed (Graham et al., 1998), for which we expected a linear and continuous improvement in both cohorts. Regarding handwriting quality, findings in past research have observed an “early plateau” in the development of ability (Graham et al., 1998; Karlsdottir and Stefansson's, 2002). However, these findings were contradictory about the earliness of this plateau during primary school. By drawing the development of handwriting quality using precise aesthetic criteria in two cohorts of different ages (coh1: Grades 2–4; coh2: Grades 3–5), the present study could help clarify the age at which this plateau is reached.

(ii) How do the orthographic complexity and graphic complexity of words influence children's spelling accuracy, handwriting speed, and handwriting quality?

With regard to orthographic complexity, past literature has demonstrated its long-lasting influence (i.e., irregular words compared to regular words) on spelling accuracy (Sprenger-Charolles et al., 2003) and on handwriting production (in children, Kandel and Perret, 2015; in adults, Palmis et al., 2019). Therefore, we expected the influence of orthographic complexity to be present at all measurement times in both cohorts. Orthographically difficult words should lead to less accurate spelling, slower handwriting, and poorer handwriting quality than orthographically simple words.

With regard to graphic complexity, past research has demonstrated that the graphomotor cost of handwriting could impact spelling accuracy (Van Reybroeck and Hupet, 2009), handwriting speed, and handwriting quality (Gosse et al., 2018). Because children in the younger cohort (coh1) had less automatised handwriting than coh2 children at the start of the study, we assumed that the impact of graphic complexity would be greater in coh1 than in coh2. Young children (coh1) should produce less accurate spelling, slower handwriting and poorer handwriting quality when words are graphically difficult than older children (coh2).

(iii) How are spelling, handwriting speed, and handwriting quality related to each other throughout development?

Overall, past research has demonstrated close associations between spelling ability and handwriting speed. In line with previous findings (Bosga-Stork et al., 2016), we expected this relationship to be stronger at the beginning of writing development and to decrease throughout primary school thanks to handwriting automatisation.

The development of handwriting quality has, to the best of our knowledge, never been investigated in association with spelling. Therefore, the hypotheses proposed in the current study are exploratory. First of all, in line with a recent experiment conducted by Arfé et al. (2020), we expect the relationship between handwriting quality and spelling to be less important than the relationship between handwriting speed and spelling accuracy. Moreover, in line with the capacity theory of writing (McCutchen's, 1996), we expect the relationship between spelling and handwriting quality to be stronger at the beginning of the study, before handwriting is automatised. Indeed, before handwriting automatisation, children need to allocate more cognitive resources to handwriting at the expense of spelling accuracy. Therefore, we assumed that the relationship between handwriting quality and spelling would be greater in coh1 (Grades 2–4) than in coh2 (Grades 3–5). This relationship should gradually decline, reflecting progressive automatisation of handwriting.

Given the evidence of a lack of association between the two components of handwriting among typically developing children (Graham et al., 1998), we expected no relation between handwriting speed and handwriting quality throughout development in either cohort.

## Methods

### Participants

Children from eight classes from two French-speaking schools in Belgium participated in the study (*N* = 136). In the present national context, cursive handwriting was the only style used at school, implying that all participants had been taught handwriting in a cursive style from the beginning of primary school. Depending on their grade at the beginning of the study, the children belonged to two different cohorts, with a year of difference between the two. Coh1 children began the study in Grade 2 and continued each year until Grade 4, and coh2 began in Grade 3 and continued each year until Grade 5. The ethical commission of the research institute (Psychological Sciences Research Institute, UCLouvain) of the main experimenter's institution approved the study (reference: Projet2016-01). The headmasters and the teachers voluntarily took part in this study, and parents' active consent was required for their child's participation. Parental consent was renewed the month preceding each testing time. Data were collected in 2017 (T1: coh1 in G2; coh2 in G3), 2018 (T2: coh1 in G3; coh2 in G4), and 2019 (T3: coh1 in G4; coh2 in G5). No selection criteria were used at the beginning of the study, but 19 children were excluded from the analysis based on the following criteria: (i) performance more than 2 SD below age-appropriate norms on at least two standardized tests (*n* =10); (ii) French as second language (*n* = 6); (iii) drop out due to moving to new schools during data collection (*n* = 3).

Therefore, the present sample was composed of 117 typically developing children with average non-verbal cognitive ability (Matrices subset of the WISC-IV, Wechsler, 2005) and receptive vocabulary ability (EVIP designation task, Dunn et al., 1993). Details regarding participants' characteristics at each measurement time are presented in Table 1. Group comparisons were conducted on all control measures at T1 (coh1: Grade 2; coh2: Grade3) based on z-scores. The independent samples *t*-test analyses revealed that both cohorts had equivalent scores in word-reading [*t*_(110)_ = −1.59; *p* = 0.115], handwriting quality [*t*_(110)_ = 1.35; *p* =0.179], and non-verbal IQ [*t*_(110)_ = −1.39; *p* = 0.160]. In contrast, the two cohorts differed on the following scores: word-spelling accuracy [*t*_(110)_ = −3.07; *p* =0.003]; handwriting speed [*t*_(110)_ = −2.85; *p* = 0.005]; and receptive vocabulary [*t*_(110)_ = −2.32; *p* =0.022].

**Table 1:** Children's scores on control measures at each measurement time.

	**Cohort 1**								
	**T1, Grade 2**, ***N*** **=** **59, 28 girls**			**T2, Grade 3**, ***N*** **=** **60, 28 girls**			**T3, Grade 4**, ***N*** **=** **57, 27 girls**		
**Control measure**	***M***	***SD***	**Range**	***M***	***SD***	**Range**	***M***	***SD***	**Range**
Age (years;months)	7;6	0.4	7;1–8;7	8;6	0.4	8;1–9;7	9;5	0.4	9;1–10;7
Non-verbal IQ	−0.32	0.95	−2.00–1.33	—	—	—	—	—	—
Vocabulary	0.38	1.32	−2.00–3.40	—	—	—	—	—	—
Spelling accuracy	−0.66	1.15	−3.84–1.14	−0.32	0.80	−1.84–1.43	−0.86	1.12	−4.32–1.65
Word reading	0.48	0.68	−0.80–2.29	0.57	0.78	−1.02–2.34	0.55	0.89	−1.51–2.08
Handwriting quality	−0.54	1.00	−2.75–1.43	−1.09	1.24	−3.8–1.89	−1.13	1.61	−3.86–2.14
Handwriting speed	0.19	0.85	−1.47–2.20	0.41	1.00	−1.32–2.59	0.26	0.88	−1.08–2.05
	**Cohort 2**								
	**T1, Grade 3**, ***N*** **=** **55, 28 girls**			**T2, Grade 4**, ***N*** **=** **56, 28 girls**			**T3, Grade 5**, ***N*** **=** **53, 26 girls**		
**Control measure**	***M***	***SD***	**Range**	***M***	***SD***	**Range**	***M***	***SD***	**Range**
Age (years;months)	8;7	0.4	8;0–9;8	9;7	0.4	9;0–10;7	10;6	0.4	10;0–11;7
Non-verbal IQ	0.12	0.93	−2.00–2.33	—	—	—	—	—	—
Vocabulary	0.87	0.94	−1.78–3.20	—	—	—	—	—	—
Spelling accuracy	−0.15	0.87	−2.56–1.55	−0.49	1.06	−4.23–1.34	−0.79	1.16	−3.47–1.34
Word reading	0.61	0.88	−0.83–2.89	0.65	0.88	−0.84–2.51	0.97	2.82	−1.37–1.98
Handwriting quality	−0.91	1.62	−5.16–1.89	−0.66	1.58	−4.43–3.57	−0.76	1.72	−6.83–2.03
Handwriting speed	0.74	1.03	−1.70–4.67	0.46	0.93	−1.47–3.05	0.84	0.86	−0.98–3.22

*The scores presented in this table are z-scores calculated by comparing children's raw scores to the clinical tests' norms for their age or grade (see Measures section for more details). These scores have a mean of 0 and a standard deviation of 1. They allow positioning children's performances in comparison to the average performances for their age or grade. Z-scores below −2 SD indicate pathological performances, and scores below −1.5 SD indicate low performances. Conversely, scores above +2 SD indicate exceptionally high performances*.

### Materials

The experimental task designed for the present study was a single-word dictation task. To complete the task, each child was given a digital tablet and a digital pen (Wacom Intuos Pro Medium and Wacom Inking Pen). The tablet size was 380 × 251 × 12 mm, with an active area of 224 × 140 mm. The single-word dictation task was composed of 40 words and remained the same at the three testing times. The words were selected according to their levels of orthographic complexity and graphic complexity.

#### Orthographic Complexity (O-simple and O-difficult)

The orthographic level of complexity refers to the spelling demand of words. To determine the words' level of orthographic complexity, we took into account word regularity and word success rate for children in Grade 2 in the French database EOLE [Echelle d'acquisition en orthographe lexicale (lexical spelling acquisition scale); Pothier and Pothier, 2003]. Regular words with a high success rate (>75% in EOLE for Grade 2) were selected for the lists of orthographically simple words (O-simple). In contrast, words with complex or irregular phoneme-grapheme correspondences and with a lower success rate (<50% in EOLE for Grade 2) were selected for the lists of orthographically difficult words (O-difficult).

#### Graphic Complexity (G-simple and G-difficult)

The graphic level of complexity refers to the graphomotor demands required in handwriting words. For example, words that include abrupt changes in the pen stroke trajectory are more complex than words containing curvy segments. This information came from a previous experiment conducted by Gosse et al. (2018), which determined how these graphic characteristics impacted children's handwriting speed and quality. Each graphic characteristic was quantified in terms of level of complexity, which led to an index giving the value of graphic complexity for each word. To determine the reference values for low and high levels of graphic complexity (G-simple vs. G-difficult), the index was applied to a complete French lexical database (LEXIQUE, New et al., 2004). Then, the 25 and 75 percentiles of the index were used as thresholds for the G-simple (index = 15.6) and G-difficult (index = 19.4) conditions.

The four lists were composed of simple or complex words on both the orthographic and graphic dimensions (O-simple, G-simple, O-difficult, and G-difficult). They were matched on the number of letters, digram frequency, success rate and graphic complexity index. Table 2 presents the four list characteristics and the 10 words composing each list.

**Table 2:** Means and standard deviations on words' characteristics per list and equivalence between lists.

	**O-simple; G-simple**		**O-difficult; G-simple**		**O-simple; G-difficult**		**O-difficult; G-difficult**	
**Words characteristics**	***M***	***SD***	***M***	***SD***	***M***	***SD***	***M***	***SD***
Number of letters	5.24^a^	0.91	5.38^ab^	0.53	6.11^ab^	0.85	6.27^b^	0.67
Digram frequency	2.52	0.35	2.85	0.38	2.81	0.29	2.60	0.32
Success rate for G2 (EOLE)	90.90^a^	8.14	12.33^b^	6.16	85.84^a^	7.91	12.91^b^	10.08
Graphic complexity index	13.24^a^	2.21	13.31^a^	1.56	22.63^b^	2.42	23.20^b^	2.81
Items	cabine carte cheval dictée gauche lire lundi papa police robe	*cabin* *card* *horse* *dictation* *left* *read* *Monday* *dad* *police* *dress*	caddie cadet coulis craie deuil gueule laitue mulot talent trait	*trolley* *cadet* *grout* *chalk* *grief* *mouth* *lettuce* *mouse* *talent* *trait*	borne branche bravo brique chambre nombre ouvrage poivre sabre score	*terminal* *branch* *cheers* *brick* *room* *number* *book* *pepper* *sword* *score*	accord annexe boxeur brebis horaire horreur kiosque membre sirop ressort	*deal* *appendix* *boxer* *ewe* *schedule* *horror* *booth* *member* *syrup* *spring*

*O-simple, orthographically simple words; O-difficult, orthographically difficult words; G-simple, graphically simple words; G-difficult, graphically difficult words; G2, Grade 2; EOLE, echelle d'acquisition en orthographe lexicale [lexical spelling acquisition scale]; Pothier and Pothier (2003). Values with different superscript letters a and b differ significantly (Bonferroni contrasts all ps < 0.001)*.

### Measures

In the present study, children were administered several control measures as well as a single-word dictation experimental task. The control measures were used to provide information about the participants' written language skills. The experimental task was designed to answer the research questions.

#### Experimental Measures

##### Spelling Accuracy in the Experimental Task

Children were given one point for each word correctly spelled and a score of 0 in the case of a spelling error. The internal reliability in the current sample given by Cronbach's alpha is 0.87. The categorical nature of spelling accuracy was taken into account in the subsequent statistical analyses.

##### Handwriting Speed in the Experimental Task

Each word's production time was directly extracted from the tablets, measured in milliseconds, giving a value for handwriting speed. The recording started at the beginning of each word's pen stroke until the end of the word. The pauses inside the words and in air phases (i.e., pen lifts) were not taken into consideration. The values for speed refer to the distance covered while the pen was on the sheet of paper expressed in centimeters per second (cm/s). A low value refers to slow handwriting, whereas high values refer to fast handwriting execution.

##### Handwriting Quality in the Experimental Task

Scoring of handwriting quality was inspired by the BHK scale (Charles et al., 2004). Five aesthetic criteria, selected because they suited the single-word context, were taken into account for handwriting quality. They all refer to a type of graphical error. Each word was assessed in a binary way, with a score of 1 assigned in the presence of a graphic abnormality and 0 being the score for the expected handwriting performance. The presence of one of the following abnormal elements was systematically noted: (1) distortion of a letter, manifesting as an abnormal letter form; (2) inconsistent letter size, referring to a situation where there is an abnormal variation in the letter size within the same word; (3) inconsistent relative height of letters, meaning that small letters (e.g., *a, i, s*) are produced at the same height as tall or tail letters (e.g., *y, t, h*) and vice versa; (4) correction of letter forms, noticeable by the presence of a graphic correction on paper; and (5) bad letter alignment within the word, when the horizontality of the handwriting is violated. For descriptive purposes, a total score per word was computed by adding the score for each criterion, leading to a maximum score of 5 per written word. For handwriting quality, high scores refer to poor handwriting, while lower scores refer to better quality. Cronbach's alphas were calculated within the present sample for each aesthetic criterion separately, and they revealed very high internal reliability: 0.92 for distortion of letters, 0.90 for inconsistent letter size, 0.85 for inconsistent relative height of letters, 0.80 for correction of letter forms, and 0.94 for bad letter alignment. For analytical purposes, the categorical nature of these five criteria was taken into account in the subsequent statistical analyses and construed as observed categorical indicators of a latent variable in all subsequent models.

#### Standardized Control Measures

##### Spelling Accuracy

Spelling ability was assessed with a sentence dictation task (Chronosdictées, Beneath et al., 2006). Children's scores were compared to the norms for their grade on phonological spelling, lexical spelling and grammatical spelling as well as their total score. The present experimental results will indicate only the children's total score.

##### Handwriting Speed in the Copying Task

Participants' handwriting speed was assessed with a standardized text copying task in the limited time of 5 min (BHK, [Concise evaluation scale for children handwriting]; Charles et al., 2004). The number of letters correctly copied was scored, which is an indicator of handwriting speed.

##### Handwriting Quality in the Copying Task

The quality of the handwriting was also assessed with the standardized test BHK (Charles et al., 2004). Each sentence was scored according to aesthetic criteria. Low scores refer to good handwriting quality, whereas high scores reflect poor handwriting. The task has a very high reported level of interrater reliability (*r* = 0.90).

##### Word Reading

Reading ability was assessed with the Lecture en Une Minute single-word reading test administered to the children [LUM, (One minute reading); Khomsi, 1999]. Children's scores were calculated by counting the number of words correctly read within 1 min. Their scores were then compared to the test's norms.

### Procedure

The longitudinal study evaluated children's spelling and handwriting performance over three years. Data were collected once a year in February–March of 2017 (T1), 2018 (T2), and 2019 (T3). At each measurement time, data collection took place over a period of 6 weeks. Each measurement time had two phases, both occurring at children's school in a quiet, empty classroom: (i) administration of the control standardized tasks and (ii) administration of the experimental task. At each measurement time, the main experimenter collaborated with two assistant experimenters for the administration of the control tasks and an engineer for the administration of the experimental task. The control tasks were administered to each child individually. Vocabulary and nonverbal IQ were only assessed at T1. The durations for the control tasks were approximately 45 min at T1 and 30 min at T2 and T3. The experimental task was administered in groups of four children. It lasted approximately 20 min. The instruction was to write down the dictated words at their usual speed. The experimenter emphasized that the dictation task would have nothing to do with their school results and that even if some words seemed too difficult for them, they should try to write them down. It was explained that their writing would be recorded inside the tablets so that we would then be able to analyse all their productions. The children were not informed about the focus on handwriting quality and speed. To have enough space to write down the 40 words, three A4 sheets for testing were provided for each child. They were attached to the top of the tablet with tape. Therefore, the testing conditions were close to how children usually write since they had to write on white paper with a Wacom ink pen. Additionally, to guide the children throughout the task and to ensure that they would write in the correct space, dashes followed by each word's determiner (either—*le* or—*la* [the] or either—*un* or—*une* [a]) were placed vertically according to the order used for the dictation. Children had to fill in the blank following each determiner. Their production was recorded in real time, with a sample frequency of 7/8 ms.

## Results

### Data Analysis

The design of the present study involved observations that were nested both within children and within words and is referred to as a stimulus sampling design (Wickham et al., 2021). While mixed effects models have been widely used to account for such cross-classified data structures (Baayen et al., 2008; Judd et al., 2012), these models are limited to univariate contexts and are limited for modeling longitudinal designs, accounting for measurement errors, and complex structural models (e.g., mediation analyses, Nestler and Back, 2017; Wickham et al., 2021). Cross-classified Bayesian structural equation modeling has been introduced to address these limitations (Muthén and Asparouhov, 2012) and offer flexible capabilities such as the inclusion of multiple observed indicators, longitudinal designs, and the inclusion of categorical indicators, as is the case in the present study.

In addition to preliminary analyses, our statistical analysis followed a five-step strategy in each cohort separately. First, Pearson bivariate correlational analyses of the scores for spelling, handwriting speed, and handwriting quality were performed. These correlational analyses were computed to quantify the strength of association or to highlight the lack of association between the standardized clinical tasks used and the experimental measures extracted from the single-word dictation task. Second, variance-component (intercept-only) models were estimated to examine the decomposition of the variance in scores between levels of analysis. Third, unconditional linear growth models were fitted to investigate the significance of time. Fourth, orthographic and graphic complexity were introduced as antecedents of our constructs' intercepts and slopes to investigate their relative predictive influence on the development of writing skills. As the final step, a unique linear growth model was fitted, which allowed us to examine the relations between skills' intercepts and slopes and their co-development over time. Preliminary analyses were conducted with IBM SPSS27, with the remainder performed using Mplus 8 with full information maximum likelihood (FIML) estimation to handle missing data.

When analyzing cross-classified structural equation models, *Mplus* uses a Bayesian approach. The use of Bayesian modeling is motivated by the precision and specificity of information about model parameters (e.g., indirect effects in mediation analysis), its performance with small samples (i.e., large sample theory is not needed), its efficiency with computationally demanding models (e.g., models with categorical indicators), and the availability of new modeling techniques (Muthén and Asparouhov, 2012). In contrast to the statistical frequentist approach, parameters in CC-BSEM are considered random variables and thus conceptualized with specific distributions. The Bayesian approach also allows researchers to determine their pre-existing beliefs about the location and dispersion of model parameters by specifying priors. Importantly, the Bayesian approach is often used for models that are not computationally possible under the traditional frequentist approach (Muthén and Asparouhov, 2012), as in the present study, and “minimally informative” prior distributions are generally preferred to limit the subjectivity in the priors that one may adopt. Previous research has shown that Bayesian estimation using relevant minimally informative prior distributions provides results similar to those of traditional modeling, such as maximum likelihood estimation (Gill, 2014). In other words, when priors are minimally informative and have a large variance, the likelihood of the data contributes more to the formation of the posterior distribution than the prior, and the estimate is therefore closer to a maximum likelihood estimate (Muthén and Asparouhov, 2012).

Model parameters in CC-BSEM were estimated using a Monte Carlo Markov chain algorithm (MCMC) with two chains as the default. Models were first performed using the minimally informative priors set by default in *Mplus*. For latent variables and observed indicators at the within level, an inverse Wishart distribution was assumed for the variance and covariance model parameters at all levels. An inverse gamma distribution was assumed for the upper-level variances of observed indicators. Threshold, loadings, intercepts, and means of latent variables were assumed to be normally distributed. When conducting Bayesian analysis, researchers are strongly invited to examine the sensitivity of their results to alternative prior distributional specifications and parameter autocorrelations with alternative thinning parameters (Muthén and Asparouhov, 2012). Given that upper-level variance components are often very close to zero and that minimally informative prior distributions might potentially impact the accuracy of these parameters in nested data structures (Browne and Draper, 2006), Wickham et al. (2021) suggested that upper-level variance components in nested designs might be better estimated using inverse gamma distributions with very small specifications. Consequently, we performed additional tests with alternative inverse gamma distributions (3, 1; 2, 1; 1, 2; 0.01, 0.01; 0.001, 0.001) for all upper-level variance components. Consistent with Wickham et al. (2021), assuming an inverse gamma distribution (0.001, 0.001) resulted in more accurate variance parameter estimates. Additional tests were also performed to examine the model sensitivity to alternative thinning parameters (i.e., 20, 50, 100). Overall, these alternative parameterizations yielded similar results and were thus not reported. The final models reported in this article are modeled with MCMC iterations set at twice the number at which the proportion scale reduction (PSR) converged, an inverse gamma distribution (0.001, 0.001) for all upper-level variance components, and thinning set by default at 1 (Muthén and Asparouhov, 2012). Although researchers are invited to examine their results using graphically represented posterior distributions (Gill, 2015), we summarized the point estimates (i.e., median) as well as credible intervals with an associated *p*-value (i.e., the credible interval does not include zero) to parallel the traditional frequentist approach and ease the interpretation process. In contrast to traditional confidence intervals, Bayesian credibility intervals (BCIs) refer to the probability regarding the credible and plausible range of estimates for the actual parameter (i.e., 95% probability that the true parameter falls within the interval). In addition, we also examined model fit indices when available: (1) the posterior predictive *p*-value (PPP), which indicates a relatively good fit when non-significant; (2) the deviance (DIC), for which the lowest values indicate better-fitting models; and (3) the averaged *R*^*2*^ across time-specific indicators.

### Preliminary Data Analysis

#### Missing Data

At the within-levels, missing data accounted for 6.60% (T1), 5.33% (T2), and 13.77% (T3) across all scores. Across cohorts and at each measurement point, missingness was present at both the word (e.g., inattention) and individual levels (e.g., absence due to illness). Using FIML estimation (Enders, 2010; Newman, 2014), all models were estimated with scores from all respondents (*N*_*coh*1_ = 2,440 and *N*_*coh*2_ = 2,240), relying on all participants who completed at least one time point. Under the missing-at-random assumption, FIML has been demonstrated to yield unbiased parameter estimates even when large amounts of longitudinal missingness are present in the data (e.g., 50%, Enders, 2010; Newman, 2014).

Distributional assumptions related to normality were examined only for speed scores, as spelling and handwriting quality scores were categorical indicators. The examination of skewness and kurtosis indices for speed yielded satisfactory values when compared to commonly reported standards (3.00 and 10.00; Kline, 2015).

#### Correlations Between Spelling, Handwriting Speed, and Quality

Pearson bivariate correlational analyses were run between children's scores in spelling, handwriting quality and handwriting speed for both the standardized tasks and the experimental task at each measurement time (coh1: G2, G3, G4; coh2: G3, G4, G5; see Tables 3A,B). The standardized measures consisted of spelling accuracy on a sentence dictation task (Chronosdictées, Beneath et al., 2006) and handwriting quality and speed scores on a text-copying task (BHK, Charles et al., 2004). The experimental task was a single-word dictation task performed on a digital tablet in which spelling accuracy, handwriting quality and speed were assessed. In the case of control measures, the scores used were the scores, obtained using test age or grade norms (i.e., z-scores). The measures extracted from the word dictation experimental task consisted of children's raw average scores at the task. For spelling, success rates for coh 1 were 37.5, 50.3, and 59.8%, for grade 2, grade 3, and grade 4, respectively. Success rates for coh 2 were 53.2, 62.9, and 71.1% for grade 3, grade 4, and grade 5, respectively. For coh1, mean handwriting quality scores were 1.70 (*SD* = 0.97), 1.47 (*SD* = 1.07), and 1.87 (*SD* = 1.09) for Grade 2, Grade 3, and Grade 4, respectively. For coh2, mean handwriting quality scores were 1.51 (*SD* = 0.86), 1.22 (*SD* = 1.01), and 1.54 (*SD* = 0.92) for Grade 3, Grade 4, and Grade 5, respectively. Mean speed scores for coh1 were 1.34 (*SD* = 0.53), 1.70 (*SD* = 0.62), and 2.07 (*SD* = 0.78) for Grade 2, Grade 3, and Grade 4, respectively. For coh2, mean speed scores were 1.63 (*SD* = 0.65), 1.70 (*SD* = 0.63), and 1.94 (*SD* = 0.71) for Grade 3, Grade 4, and Grade 5, respectively. More details regarding children's performances at the experimental word-dictation task in relation to orthographic and graphic levels of complexity can be found in the Appendix section.

**Table 3:** Correlations between experimental and control measures of spelling and handwriting abilities at the three measurement times in cohort 1 and cohort 2.

**Measure**	**1**	**2**	**3**	**4**	**5**	**6**	**7**	**8**	**9**	**10**	**11**	**12**	**13**	**14**	**15**	**16**	**17**
**COHORT 1**																	
**Experimental single-word dictation task**																	
1. Spelling accuracy G2	–																
2. Spelling accuracy G3	0.650^**^	–															
3. Spelling accuracy G4	0.566^**^	0.748^**^	–														
4. Handwriting speed G2	−0.079	−0.1	−0.031	–													
5. Handwriting speed G3	−0.036	0.055	0.021	0.576^**^	–												
6. Handwriting speed G4	−0.079	0.027	0.062	0.27	0.508^**^	–											
7. Handwriting quality G2^a^	0.085	−0.174	0.012	0.285^*^	0.183	0.014	–										
8. Handwriting Quality G3^a^	−0.141	−0.140	0.026	0.215	0.204	0.178	0.693^**^	–									
9. Handwriting quality G4^a^	−0.205	−0.400^**^	−0.226	0.156	0.076	0.356^*^	0.542^**^	0.652^**^	–								
**Standardized spelling task (Chronosdictée)**^**b**^																	
10. Spelling accuracy G2	0.615^**^	0.650^**^	0.511^**^	−0.056	−0.056	0.048	−0.291^*^	−0.491^**^	−0.356^*^	–							
11. Spelling accuracy G3	0.600^**^	0.727^**^	0.711^**^	0.136	0.111	0.248	−0.077	−0.120	−0.221	0.546^**^	–						
12. Spelling accuracy G4	0.504^**^	0.705^**^	0.772^**^	0.166	0.165	0.191	0.055	0.083	−0.186	0.458^**^	0.762^**^	–					
**Standardized handwriting copying task (BHK)**^**b**^																	
13. Handwriting speed G2	0.299^*^	0.299^*^	0.483^**^	0.293^*^	0.289^*^	0.099	−0.072	−0.070	0.036	0.297^*^	0.309^*^	0.236	–				
14. Handwriting speed G3	0.180	0.244	0.369^**^	0.341^*^	0.341^*^	0.212	−0.012	−0.006	−0.001	0.051	0.433^**^	0.221	0.695^**^	–			
15. Handwriting speed G4	0.145	0.235	0.491^**^	0.349^*^	0.258	0.316^*^	0.173	0.181	0.069	0.041	0.404^**^	0.399^**^	0.611^**^	0.717^**^	–		
16. Handwriting quality G2^a^	−0.065	−0.221	−0.214	0.066	−0.192	−0.252	0.403^**^	0.358^**^	0.474^**^	−0.339^*^	−0.145	−0.194	−0.057	−0.111	−0.185	–	
17. Handwriting quality G3^a^	−0.023	−0.276^*^	−0.222	0.044	0.063	−0.085	0.492^**^	0.636^**^	0.579^**^	−0.365^**^	−0.377^**^	−0.260	0.079	−0.059	−0.053	−0.558^**^	–
18. Handwriting quality G4^a^	−0.015	−0.236	−0.086	0.047	0.059	0.277	0.530^**^	0.591^**^	0.695^**^	−0.239	−0.070	−0.006	−0.047	−0.047	0.194	−0.351^*^	−0.427^**^
**COHORT 2**																	
**Experimental single-word dictation task**																	
1. Spelling accuracy G3	–																
2. Spelling accuracy G4	0.840^**^	–															
3. Spelling accuracy G5	0.812^**^	0.794^**^	–														
4. Handwriting speed G3	0.280^*^	0.295^*^	0.255	–													
5. Handwriting speed G4	0.159	0.16	0.264	0.580^**^	–												
6. Handwriting speed G5	−0.109	−0.042	0.062	0.529^**^	0.686^**^	–											
7. Handwriting quality G3^a^	−0.058	0.113	−0.029	0.317^*^	0.012	0.115	–										
8. Handwriting quality G4^a^	0.104	0.148	0.147	0.236	0.13	0.228	0.305^*^	–									
9. Handwriting quality G5^a^	−0.299^*^	−0.259	−0.188	0.145	0.114	0.410^**^	0.455^**^	0.443^**^	–								
**Standardized spelling task (Chronosdictée)**^**b**^																	
10. Spelling accuracy G3	0.782^**^	0.758^**^	0.746^**^	0.277^*^	0.259	0.079	−0.061	0.071	−0.265	–							
11. Spelling accuracy G4	0.753^**^	0.733^**^	0.701^**^	0.19	0.222	0.037	0.052	0.097	−0.254	0.802^**^	–						
12. Spelling accuracy G5	0.729^**^	0.730^**^	0.702^**^	0.178	0.286^*^	0.079	−0.061	0.078	−0.239	0.726^**^	0.835^**^	–					
**Standardized handwriting copying task (BHK)**^**b**^																	
13. Handwriting speed G3	0.276^*^	0.252	0.26	0.335^*^	0.314^*^	0.163	−0.095	−0.116	−0.168	0.379^**^	0.353^*^	0.336^*^	–				
14. Handwriting speed G4	0.274^*^	0.163	0.191	0.105	0.276^*^	−0.032	0.008	−0.072	−0.241	0.299^*^	0.333^*^	0.437^**^	0.622^**^	–			
15. Handwriting speed G5	0.531^**^	0.505^**^	0.518^**^	0.146	0.108	0.047	0.001	0.101	−0.123	0.498^**^	0.629^**^	0.618^**^	0.633^**^	0.625^**^	–		
16. Handwriting quality G3^a^	−0.219	−0.078	−0.177	0.316^*^	0.000	0.188	0.754^**^	0.290^*^	0.603^**^	−0.142	−0.112	−0.203	−0.104	−0.193	−0.147	–	
17. Handwriting quality G4^a^	−0.142	−0.109	0.035	0.034	0.141	0.206	0.216	0.549^**^	0.365^**^	−0.026	−0.031	0.006	−0.190	−0.041	0.011	−0.372^**^	–
18. Handwriting quality G5^a^	−0.004	0.143	0.153	0.228	0.108	0.358^**^	0.467^**^	0.482^**^	0.474^**^	0.043	0.052	0.057	−0.098	−0.230	−0.004	−0.691^**^	0.447^**^

* *p < 0.05*,

** *p < 0.01*.

a *The correlations involving the handwriting quality scores should be interpreted keeping in mind that a high score refers to a high number of graphical errors, and a low score refers to good handwriting quality*.

b *Z-scores given by comparison to test norms were used for Chronosdictée and BHK*.

First, one can notice that for the same ability, namely, spelling, and handwriting quality, measures from the standardized tasks and the experimental task were strongly correlated (e.g., for spelling, all *r*s > 0.504 and all *p*s < 0.01 in Cohort 1, see Table 3A; all *r*s > 0.701 and all *p*s < 0.01 in Cohort 2, see Table 3B). This demonstrates that even if the tasks were different (i.e., sentence dictation for standardized spelling measure vs. single-word dictation for experimental spelling measure), they measured similar abilities. Concerning handwriting speed, measures from the standardized task and from the experimental task (i.e., number of words copied in 5 min for handwriting speed standardized measure vs. speed of graphomotor execution in cm/s for the experimental measure) were not as strongly correlated. However, handwriting speed in the standardized task considers pauses during writing and was measured at the text level. In contrast, handwriting speed in the experimental task specifically measured graphomotor execution at the word level, without taking pauses into account. Finally, in a great majority of cases, the correlations for the same ability were significant over time (i.e., between T1 and T2, T2 and T3, and T1 and T3).

The analyses highlighted significant correlations between spelling accuracy and handwriting speed in both cohorts, indicating that spelling and speed were positively related throughout development (e.g., correlations between standardized spelling scores and standardized handwriting speed scores in Cohort 1 *r* = 0.297, *p* < 0.05 in Grade 2*, r* = 0.433*, p* < 0.01 in Grade 3 and *r* = 0.399, *p* < 0.01 in Grade 4, see Table 3A; in Cohort 2 *r* = 0.379, *p* < 0.01 in Grade 3, *r* = 0.333, *p* < 0.05 in Grade 4 and *r* = 0.618 *p* < 0.01 in Grade 5, see Table 3B). Second, the correlations demonstrated a negative relation between speed and handwriting quality in the experimental word dictation task in both cohorts, revealing that fast handwriting was associated with greater numbers of graphic errors (e.g., in Cohort 1 in Grade 4: *r* = 0.365, *p* < 0.05, see Table 3A; in Cohort 2 in Grade 5: *r* = 0.410, *p* < 0.01, see Table 3B).

### Cross-classified Growth Curve Models

#### Variance Components

Variance component models (intercept-only models) were fitted to investigate intraclass correlation coefficients (ICCs) across levels of analysis. For spelling, most variability was found at the word level (ICC_word_ = 0.82 and 0.77 for coh1 and coh2) rather than at the individual level (ICC_child_ = 0.09 and 0.12 for coh1 and coh2). This pattern of results suggests that variability is due to differences between words rather than differences between individuals. For speed, a fair amount of variability was found both at the word (ICC_word_ = 0.40 and 0.17 for coh1 and coh2) and the individual level (ICC_child_ = 0.50 and 0.77 for coh1 and coh2). Finally, significant variance components were found at the word (ICC_word_ = 0.38 and 0.46 for coh1 and coh2) and the individual level (ICC_child_ = 0.53 and 0.38 for coh1 and coh2) for handwriting quality. Importantly, all variance components were found significant at the word and individual level. For spelling, the intercept-only model explained similar variance both at the word (*R*^*2*^_word_ = 0.78 and 0.89 for coh1 and coh2) and at the individual level (*R*^*2*^_child_ = 0.80 and 0.90 for coh1 and coh2). For speed, the model explained more variance at the word level (*R*^*2*^_word_ = 0.81 and 0.84 for coh1 and coh2) than at the individual level (*R*^*2*^_child_ = 0.24 and 0.61 for coh1 and coh2). Similarly, the model explained more variance at the word level (*R*^*2*^_word_ = 0.95 and 0.96 for coh1 and coh2) than at the individual level for handwriting quality (*R*^*2*^_child_ = 0.76 and 0.62 for coh1 and coh2).

#### Unconditional Linear Growth Models

To test the significance of time, unconditional linear growth curve models were fitted for each construct and for each cohort separately (see Table 4). For spelling, the results showed in both cohorts that a linear trend fit the data well, as indicated by their non-significant PPP. In both cohorts, fixed effects were identified at the individual level and showed that children tended to improve in spelling over time, though children in coh2 displayed a weaker trend in improvement (*B* = 0.84 [0.64; 1.04] and 0.63 [0.43; 0.80] for coh1 and coh2). Compared to the variance component model, this unconditional linear growth model explained an increase of approximately 19.00 and 4.47% of the total variance for coh1 at the word and individual levels, respectively. In contrast, the model only explained an increase of approximately 7.86 and 2.63% of the total variance in coh2 at the word and individual levels, respectively. For speed, the model also fit the data well, as indicated by non-significant PPPs and a decreased deviation statistic compared to that for the intercept-only model. Children displayed a significant increase in speed over time, with children in coh2 having a lower positive trend than children in coh1 (*B* = 0.35 [0.26; 0.44] and 0.16 [0.10; 0.23] for coh1 and coh2). When examining the change in total variance explained compared to that in variance component models, these results showed that the addition of a linear trend explained an increase of approximately 0.44 and 23.74% of the total variance for coh1 at the word and individual levels, respectively. In coh2, the linear growth model explained an increase of approximately 2.90 and 7.93% at the word and individual levels, respectively. For handwriting quality, the linear growth models fit the data poorly, as indicated by significant PPPs in both cohorts, and suggested that no linear change occurred over time (*B* = 0.06 [−0.03; 0.16] and 0.04 [−0.04; 0.11] for coh1 and coh2). In coh1, the addition of a linear trend explained increases of 1.04 and 4.10% at the word and individual levels, respectively. In coh2, these values were 0.30 and 11.60% at the word and individual levels, respectively. In all models, all random variance components were significant at both the word and individual levels, suggesting that predictors of intercepts and slopes are likely to account for some of the variance components in all three constructs.

**Table 4:** Estimates of the unconditional linear growth curve models for spelling accuracy, handwriting speed, and handwriting quality.

	**Cohort 1**			**Cohort 2**		
	**Spelling**	**Speed**	**Quality**	**Spelling**	**Speed**	**Quality**
	**Estimate [95% BCI]**	**Estimate [95% BCI]**	**Estimate [95% BCI]**	**Estimate [95% BCI]**	**Estimate [95% BCI]**	**Estimate [95% BCI]**
**Fixed effects**						
Intercept_child_	0.00 [0.00; 0.00]	1.34 [1.22; 1.46]	0.00 [0.00; 0.00]	0.00 [0.00; 0.00]	1.63 [1.48; 1.78]	0.00 [0.00; 0.00]
Slope_child_	0.84 [0.64; 1.04]^*^	0.35 [0.26; 0.44]^*^	0.06 [−0.03;0.16]	0.63 [0.43; 0.80]^*^	0.16 [0.10; 0.23]^*^	0.04 [−0.04;0.11]
**Random effects**						
Intercept_child_	0.32 [0.13; 0.65]^*^	0.09 [0.02; 0.18]^*^	0.11 [0.05; 0.21]^*^	0.62 [0.37; 1.05]^*^	0.17 [0.08; 0.30]^*^	0.06 [0.02; 0.13]^*^
Slope_child_	0.03 [0.00; 0.13]^*^	0.02 [0.00; 0.08]^*^	0.01 [0.00; 0.03]^*^	0.01 [0.00; 0.04]^*^	0.01 [0.00; 0.05]^*^	0.00 [0.00; 0.02]^*^
Intercept_word_	4.70 [2.79; 8,30]^*^	0.02 [0.01; 0.04]^*^	0.07 [0.04; 0.13]^*^	3.98 [2.40; 6.95]^*^	0.05 [0.03; 0.08]^*^	0.05 [0.02; 0.11]^*^
Slope_word_	0.03 [0.00; 0.16]^*^	0.01 [0.00; 0.01]^*^	0.00 [0.00; 0.01]^*^	0.07 [0.00; 0.24]^*^	0.00 [0.00; 0.00]^*^	0.00 [0.00; 0.01]^*^
Intercept_residual_	0.52 [0.13; 1,20]^*^	0.02 [0.01; 0.03]^*^	0.02 [0.00; 0.06]^*^	0.54 [0.21; 1.04]^*^	0.01 [0.01; 0.02]^*^	0.06 [0.03; 0.12]^*^
Slope_residual_	0.69 [0.30; 1,14]^*^	0.01 [0.01; 0.02]^*^	0.02 [0.00; 0.05]^*^	0.26 [0.07; 0.51]^*^	0.00 [0.00; 0.01]^*^	0.02 [0.00; 0.04]^*^
***PPP***	0.40	0.31	0.00	0.45	0.34	0.00
***DIC***	–	7,050.80	–	–	5,935.02	–
***R***^*2*^_**child**_	0.85	0.47	0.80	0.93	0.69	0.74
***R**^*2*^*_**word**_	0.97	0.82	0.96	0.97	0.87	0.97
**Number of words**	40	40	40	40	40	40
**Number of children**	61	61	61	56	56	56
**Number of observations**	2,440	2,440	2,440	2,240	2,240	2,240
**Number of parameters**	17	20	66	17	20	66

*R-square averaged across time points. PPP, posterior predictive p-value; DIC, deviance; BCI, Bayesian credibility interval*.

* *p < 0.05*.

#### Conditional Linear Growth Curve Model of Orthographic and Graphic Complexity

From these linear growth curve models, we added the orthographic and graphic complexity—as well as their interaction—as predictors of intercept and slope terms for each construct separately (see Tables 5, 6). As these two variables are word-specific predictors, random effects were allowed at the within-word levels, and fixed effects were identified only at the word level. These analyses yielded contrasting results for each construct. For spelling, orthographic and graphic complexity—as well as their interaction—were significant predictors of the intercept in coh1 (i.e., Grade 2). This result suggests that words that are both orthographically and graphically complex are spelled the worst. In coh2, only orthographic complexity was found to be a predictor of the intercept of spelling (i.e., Grade 3). None of the predictors were related to the development of spelling over time. For speed, contrasting results were also found when comparing coh1 and coh2. In coh1, whereas neither orthographic nor graphic complexity was found to be a predictor of the initial level of speed, graphic complexity significantly predicted the development of speed (*B* = −0.10 [−0.17; −0.03]). In coh2, graphic complexity predicted the initial levels (i.e., grade 3; *B* = −0.19 [−0.33; −0.04]) but did not predict the development of speed over time. For handwriting quality, similar results were found among coh1 and coh2. The results showed that only graphic complexity was a predictor of the initial levels of handwriting quality in both cohorts (i.e., coh1: *B* = 0.35 [0.18; 0.54]; coh2: *B* = 0.25 [0.03; 0.40]).

**Table 5:** Estimates of the conditional linear growth curve models of spelling, handwriting speed, and handwriting quality on orthographic and graphic complexity in cohort 1.

	**Spelling accuracy**		**Handwriting speed**		**Handwriting quality**	
	**Intercept**	**Slope**	**Intercept**	**Slope**	**Intercept**	**Slope**
**FIXED EFFECTS**						
Orthographic complexity	−3.97 [−4.92; −3.37]^*^	0.34 [−0.13; 0.83]	−0.06 [−0.17; 0.05]	−0.05 [−0.12; 0.02]	−0.15 [−0.33; 0.02]	−0.02 [−0.12; 0.07]
Graphic complexity	−0.92 [−1.42; −0.41]^*^	0.16 [−0.27; 0.42]	−0.04 [−0.17; 0.08]	−0.10 [−0.17; −0.03]^*^	0.35 [0.18; 0.54]^*^	0.00 [−0.10; 0.07]
Interaction	1.22 [0.11; 2.35]^*^	−0.33 [−0.79; 0.21]	−0.11 [−0.30; 0.15]	0.09 [−0.03; 0.23]	−0.06 [−0.40; 0.26]	−0.12 [−0.30; 0.07]
**RANDOM EFFECTS**						
Orthographic complexity_word_	0.07 [0.00; 1.88]^*^	0.01 [0.00; 0.24]^*^	0.01 [0.00; 0.08]^*^	0.00 [0.00; 0.02]^*^	0.02 [0.00; 0.17]^*^	0.00 [0.00; 0.02]^*^
Graphic complexity_word_	0.12 [0.00; 1.88]^*^	0.01 [0.00; 0.29]^*^	0.01 [0.00; 0.07]^*^	0.00 [0.00; 0.02]^*^	0.02 [0.00; 0.17]^*^	0.00 [0.00; 0.02]^*^
Interaction_word_	0.20 [0.00; 7.11]^*^	0.02 [0.00; 0.92]^*^	0.01 [0.00; 0.07]^*^	0.01 [0.00; 0.07]^*^	0.04 [0.02; 0.65]^*^	0.01 [0.00; 0.07]^*^

*For handwriting quality scores, a high score refers to poor handwriting, and a low score refers to good quality*.

* *p < 0.05*.

**Table 6:** Estimates of the conditional linear growth curve models of spelling, handwriting speed, and handwriting quality on orthographic and graphic complexity in cohort 2.

	**Spelling accuracy**		**Handwriting speed**		**Handwriting quality**	
	**Intercept**	**Slope**	**Intercept**	**Slope**	**Intercept**	**Slope**
**FIXED EFFECTS**						
Orthographic complexity	−3.54 [−4.25; −2.95]^*^	−0.28 [−0.08; 0.73]	−0.13 [−0.26; 0.00]	0.04 [−0.01; 0.09]	−0.11 [−0.29; 0.08]	−0.03 [−0.12; 0.06]
Graphic complexity	−0.45 [−1.11; 0.04]	0.18 [−0.13; 0.55]	−0.19 [−0.33; −0.04]^*^	0.02 [−0.03; 0.06]	0.25 [0.03; 0.40]^*^	0.06 [−0.04; 0.15]
Interaction	0.65 [−0.35; 1.84]	−0.43 [−1.15; 0.17]	0.10 [−0.16; 0.36]	−0.01 [−0.11; 0.08]	−0.11 [−0.47; 0.27]	−0.02 [−0.18; 0.19]
**RANDOM EFFECTS**						
Orthographic complexity_word_	0.11 [0.00; 2.20]^*^	0.02 [0.00; 0.46]^*^	0.01 [0.00; 0.13]^*^	0.00 [0.00; 0.01]^*^	0.03 [0.00; 0.19]^*^	0.00 [0.00; 0.02]^*^
Graphic complexity_word_	0.03 [0.00; 1.60]^*^	0.04 [0.00; 0.59]^*^	0.02 [0.00; 0.14]^*^	0.00 [0.00; 0.01]^*^	0.01 [0.00; 0.18]^*^	0.00 [0.00; 0.02]^*^
Interaction_word_	0.23 [0.00; 8.78]^*^	0.07 [0.00; 1.81]^*^	0.02 [0.02; 0.44]^*^	0.00 [0.00; 0.03]^*^	0.06 [0.01; 0.70]^*^	0.01 [0.00; 0.05]^*^

*For handwriting quality scores, a high score refers to poor handwriting, and a low score refers to good quality*.

* *p < 0.05*.

#### Concurrent Development of Spelling, Handwriting Speed, and Handwriting Quality

The last step of the present analyses was to investigate the developmental relations between the three constructs over time. For each cohort, we fitted a unique model with the three unconditional linear growth models in each cohort separately (see Table 7). Before examining the relations between constructs' intercepts and slopes, means and variances of intercept and slopes were examined and were found to be strictly similar to the results previously identified. In coh1, only the correlation between the intercept of speed and its slope was found to be significant. Compared to children with low speed at Grade 2, this suggests that children with a higher speed exhibited a lower positive trend over time. In coh2, the results showed several significant correlations between the constructs' intercepts and slopes. First, the initial levels of speed were related to the initial levels of handwriting quality (*r* = 0.078 [0.01;0.018]) and initial levels of spelling (*r* = 0.16 [0.01;0.36]). However, the initial levels of spelling and handwriting quality were not related. Second, the initial levels of spelling were significantly related to the development of speed over time (*r* = −0.09 [−0.20; −0.02]). This finding suggested that children with poorer levels of spelling at Grade 3 tended to exhibit a greater increase in speed over time than children with better spelling at Grade 3. Overall, these results suggested that the improvements in spelling, speed and handwriting quality were rather unrelated, as none of the correlations between the constructs' slopes were found to be significant.

**Table 7:** Correlation coefficients at the individual level between growth factors of spelling, handwriting speed, and handwriting quality in cohort 1 and cohort 2.

	**Spelling**		**Speed**		**Quality**	
	**Intercept**	**Slope**	**Intercept**	**Slope**	**Intercept**	**Slope**
**SPELLING**						
Intercept	–	−0.035	0.156^*^	−0.094^*^	−0.003	−0.037
Slope	0.000	–	0.016	0.014	0.006	0.002
**SPEED**						
Intercept	−0.011	0.010	–	−0.024	0.078^*^	−0.011
Slope	−0.005	−0.004	−0.043^*^	–	−0.010	0.013
**QUALITY**						
Intercept	−0.020	−0.031	0.047	−0.027	–	−0.012
Slope	−0.032	−0.002	−0.010	0.023	−0.015	–

*Correlations for coh1 (Grades 2–4) are below the diagonal, and correlations for coh2 (Grades−5) are above the diagonal. For handwriting quality scores, a high score refers to poor handwriting, and a low score refers to good quality*.

* *p < 0.05*.

## Discussion

The present study addressed the question of the development of writing abilities at the word level by assessing for the first time in a longitudinal study the two transcription skills with measure of spelling accuracy, handwriting speed, and handwriting quality. One hundred and seventeen French-speaking children were assessed once a year for three consecutive years (coh1: Grades 2–4; coh2: Grades 3–5). They performed a single-word dictation task composed of words that varied in orthographic and graphic complexity. Using a CC-BSEM approach, linear growth curve models were conducted in each cohort separately in order to investigate the longitudinal development of the transcription skills.

### Key Findings

This study is the first longitudinal study in which the development of spelling, handwriting speed, and handwriting quality was investigated concurrently. The results revealed that word spelling and handwriting speed continued to improve until the end of the study (Grade 5), indicating that these skills have not yet reached a mature level in the later years of primary school. Conversely, the results revealed no growth in handwriting quality during the study. This last finding is a valuable contribution to the field of research in writing development, since the results regarding the age at which children reach a plateau in handwriting quality were contradictory. Our longitudinal data indicate that the plateau in this ability had already occurred before the start of the study, i.e., before mid-Grade 2, suggesting that, in a single word writing context, handwriting quality development is limited to the very beginning of primary school.

Thanks to the cross-classified structural equation modeling analysis, this study adopted a psycholinguistic approach allowing the investigation of the impact of orthographic and graphic complexity on spelling and handwriting performances throughout development. Novel findings were revealed in relation to the graphic complexity of words. Children's writing performance was impacted by graphic complexity, with handwriting being slower for graphically difficult words. Graphic complexity even impacted the development of handwriting speed in the young cohort (coh1: Grades 2–4): children's improvement in speed was greater for graphically simple words than for graphically complex words. Moreover, graphic complexity also impacted spelling outcomes, but this effect was limited to the beginning of the study (Grade 2). To the best of our knowledge, this is the first study with a large sample of typically developing children showing that the graphomotor cost can impede spelling in beginning writers. The orthographic complexity of words had a significant influence on spelling outcomes in both cohorts at the start of the study, with irregular complex words being produced less accurately. This gap in performance between orthographically difficult and simple words (i.e., irregular and regular words) was present until the end of the study (Grade 5). Orthographic complexity did not significantly impact handwriting outcomes, with orthographically difficult words being produced as legibly and as fast as orthographically simple words. Finally, this longitudinal study investigated how the three writing abilities (spelling, handwriting speed and quality) were related to each other. This study demonstrated a reverse relationship between the two components of handwriting, namely, speed and quality, indicating that fast handwriting is associated with less legible handwriting. While past research has concluded that speed and legibility (i.e., measure of handwriting quality using five aesthetic criteria in the current study) are rather independent from each other, this experiment suggests a significant association between these two components of handwriting. Moreover, positive associations between handwriting speed and spelling accuracy were demonstrated in the present sample, indicating that children who write fast are also those with strong spelling ability.

### Development of the Transcription Skills: Spelling Accuracy, Handwriting Speed, and Handwriting Quality

The first aim of the study was to examine the development of the three variables separately. The present study revealed that children's spelling accuracy and handwriting speed continued to improve during primary school. Throughout the three measurement times, (coh1: Grade 2 to 4; coh2: Grade 3 to 5), spelling and speed significantly improved, and their developmental trajectories followed a positive linear trend. These findings are in line with our predictions and congruent with previous findings (Graham et al., 1998; Alves and Limpo, 2015). Notably, the slope estimates indicated that the improvement in both spelling and speed is more important between Grade 2 and Grade 4 (coh1) than between Grade 3 and Grade 5 (coh2). Conversely, the longitudinal data did not reveal improvement in handwriting quality meaning that it did not change significantly over time in the two cohorts of children (coh1: Grade 2–4; coh2: Grade 3–5). Although rarely studied, this result for handwriting quality is in line with previous findings. An early plateau at the end of Grade 1 was reported in a longitudinal experiment conducted by Karlsdottir and Stefansson's (2002). An explanation for these findings could be the lack of explicit handwriting instruction after Grade 1 in the Belgian context of the present study. By beginning the data collection in the second semester of Grade 2 (coh1) and Grade 3 (coh2), the present study may have not targeted the critical period for improving handwriting quality In other words, our results suggest that the temporal window during which handwriting quality develops occurred before the start of our study, between the beginning of Grade 1 and the second semester of Grade 2.

### Effect of Word Orthographic and Graphic Complexity on Writing Development

The second aim of the study was to evaluate the influence of the orthographic and graphic complexity of words, both on the initial levels of transcription abilities at the start of the study and on the pace of development of these abilities. The experimental dictation task was composed of words that were either orthographically simple, i.e., regular words, or orthographically difficult, i.e., irregular words. The same manipulation was used for graphic complexity, with words being either graphically simple or graphically difficult, i.e., containing more abrupt changes in the pen stroke trajectory (Gosse et al., 2018).

The orthographic complexity of words revealed a significant influence on spelling outcomes. The results showed that the orthographic complexity of words was predictive of children's initial level of spelling accuracy in both cohorts (coh1: Grade 2; coh2: Grade 3). This result is not surprising, since the dictation task was built to contrast regular words with inconsistent words, the latter leading to a high percentage of spelling errors. Notably, at the end of the experiment, children in Grade 5 were spelling accurately <50% of the orthographically complex words, while over 90% of the regular words were spelled correctly. This finding is in line with past research in which the long lasting impact of word regularity had been highlighted (Sprenger-Charolles et al., 2003). The results demonstrated that orthographic complexity did not explain the variability in handwriting skills during the single-word dictation task, both speed and quality, between children at the start of the study, whether in Grade 2 (coh1) or in Grade 3 (coh3). These findings suggest that handwriting quality and speed are invariant whether words are irregular or regular, which is inconsistent with the results found for children aged 8–11 by Kandel and Perret (2015) in a single-word copying task, and with our predictions. A plausible explanation for the absence of influence on speed could lie in the measure of speed used in the present study, which was an actual measure of motor execution in terms of the distance covered by seconds (cm/s). This method of measuring speed did not include pauses and latency. We can hypothesize that, like in past experiments, orthographically complex words may have led children to think more about spelling before starting handwriting (Kandel and Perret, 2015) and that pauses may have been more frequent during handwriting for irregular words than for simple regular words (Sumner et al., 2013). Measures of latency before writing or pauses during writing would have been useful to confirm this hypothesis. Moreover, one should recall that the experimental task was a single-word dictation task with no time constraint. Therefore, children could take as much time as needed to think before writing and to execute handwriting. It is plausible that in a different handwriting task with a time constraint, the challenging spelling of irregular words would have led to poorer handwriting quality, as would be predicted by the capacity theory of writing (McCutchen's, 1996). Our results did not show any significant impact of orthographic complexity on the way each ability improved. Spelling, handwriting speed, and handwriting quality developed to the same extent whether words were simple (i.e., regular words) or difficult (i.e., irregular words). This result suggests that the gap in performance between orthographically simple and complex words observed at T1 for spelling accuracy in both cohorts remained constant throughout the study, suggesting that the regularity effect is of similar importance from Grade 2 to Grade 5.

The graphic complexity of words revealed numerous significant influences on children's performance in handwriting speed, spelling and handwriting quality and on their development. Regarding handwriting speed, the results differed between the two cohorts. At the beginning of the study, graphic complexity significantly explained handwriting speed in the older cohort (coh2: Grade 3), whereas it was not a predictor of handwriting speed in the young cohort (coh1: Grade 2). The expected impact of graphic complexity on handwriting speed was revealed in the older cohort (Grade 3), who had more automatised handwriting skills. This finding suggests that when children have a certain level of graphomotor automatisation, handwriting speed starts to vary according to graphic complexity, with graphically simple words leading to faster handwriting than graphically difficult words. The finding that the graphic complexity of words did not impact handwriting speed in Grade 2 can be explained by children's young age and limited experience in handwriting at the start of the study. In Grade 2, children may have been invariably slow at handwriting execution, whether words were graphically simple or complex. Consequently, graphic complexity did not play a role at this early stage of development. This assumption is in line with previous findings in a study comparing handwriting speed in children with dyslexia and young typically developing peers in Grade 2 (Gosse and Van Reybroeck, 2020). Regarding the developmental pace of handwriting speed, the effect of graphic complexity also differed depending on the cohort. The increase in handwriting speed was significantly related to graphic complexity in the younger cohort (coh1: Grades 2–4) but not in the more experienced cohort (coh2: Grades 3–5). In the younger cohort, handwriting speed developed faster for graphically simple words than for graphically difficult words. As presented above, these young coh1 children did not initially (Grade 2) present faster handwriting for graphically simple words compared to difficult words. However, the impact of graphic complexity on handwriting speed seems to have emerged during the study, since the pace of development varied depending on graphic complexity. Conversely, the handwriting speed of coh2 children improved equally, regardless of the graphic complexity of words. This reveals that the gap in speed performance observed in coh2 at the start of the study between graphically simple and graphically difficult words remained stable over time (coh2: Grades 3–5). In the case of children with more automatised graphomotor abilities, as we assume was the case for children in Grade 3 at the start of the study (coh2), the impact of graphic complexity on handwriting speed was already present at the start of the study and remained stable over time (Grades 3–5). Interestingly, while handwriting speed has often been interpreted as a reflection of orthographic processes (Sumner et al., 2013; Kandel and Perret, 2015; Palmis et al., 2019), this study provides a novel finding regarding the effect of graphic complexity on speed.

Regarding spelling accuracy, graphic complexity explained a significant portion of the performance variability among children at the start of the study in the young cohort (Grade 2). Moreover, the interaction between orthographic and graphic complexity was significant, with difficult words leading to less accurate spelling in beginning writers in Grade 2. Conversely, this effect of graphic complexity was not present in the older cohort, who started in Grade 3. In other words, children in Grade 2 spelled graphically complex words less accurately than graphically simple words, while children in Grade 3 were not impacted by the manipulation of graphic complexity. This different finding can potentially be explained by the capacity theory of writing (McCutchen's, 1996). Indeed, young children (coh1) had to recruit more cognitive resources for graphomotor processes than coh2 children, since their handwriting abilities were not as mature and automatised. It can then be assumed that in coh1 (Grade 2), the increasing graphic complexity of words required children to allocate more resources to the handwriting processes at the expense of spelling. Moreover, the results showed that the development of spelling over time did not depend on graphic complexity. Children from the two cohorts improved spelling accuracy over time to the same extent whether words were graphically simple or complex.

Regarding handwriting quality the findings highlighted in both cohorts a significant impact of graphic complexity on children's initial performance (coh1: Grade 2; coh2: Grade 3). Not surprisingly, graphically difficult words led to poorer handwriting quality than graphically simple words (Gosse et al., 2018). The results showed that graphic complexity had no significant impact on the development of handwriting quality. This means that handwriting quality developed at the same pace for graphically simple and difficult words. These results indicated that the impact of graphic complexity on handwriting quality remained stable until the end of the study (coh1: Grade 4; coh2: Grade 5), with graphically difficult words still being produced less legibly than graphically simple words for the oldest children. These findings are novel, since handwriting quality has only rarely been investigated (Barnett et al., 2018; Gosse et al., 2018; Caravolas et al., 2020) and, to the best of our knowledge, never been studied in relation to graphic complexity.

### Relations Between Spelling, Handwriting Speed, and Handwriting Quality

The last objective of the present study was to examine the developmental relations between spelling, handwriting speed, and handwriting quality to shed light on the potential reciprocal influences between transcription skills. In addition, the correlational analyses allowed us to further refine our understanding of the relationships between transcription skills. First, while spelling and handwriting speed showed continuous development until the end of the study, our analyses did not reveal a significant impact of the improvement in one skill on the improvement in the other. This is visible in the absence of significant correlations between the slopes for each skill. In other words, the development of spelling was not explained by the development of speed, and handwriting speed improvement was not explained by the improvement in spelling accuracy.

The results revealed a significant association between spelling accuracy and handwriting speed in coh2 (Grade 3–5), with spelling in Grade 3 influencing both the initial level and the development of speed. The analyses showed that the pace of development of speed was significantly related to spelling performance at the start of the study (Grade 3). This means that poor spellers in Grade 3 improved their speed of handwriting to a greater extent than children with higher spelling accuracy. To interpret this finding, it is interesting to add that correlational analyses revealed a positive association between spelling accuracy and handwriting speed in both cohorts throughout development (coh1: Grades 2–4; coh2: Grades 3–5). It can then be assumed that the poor spellers in Grade 3 were also slower at handwriting. Therefore, the poor spellers, also slow at handwriting, possibly had more room for improving their handwriting speed than the better spellers.

A last significant finding from the analyses concerns the relationship between handwriting speed and handwriting quality in Grade 3 (coh2). The analyses revealed that the initial levels of handwriting speed and handwriting quality were negatively related. This means that the fast children were the ones with poorer handwriting quality in Grade 3, as measured by the presence of aesthetic errors. However, it is important to remind that a word containing aesthetic errors in the present study did not indicate legibility issues, as handwriting can still be easily decoded even when the word is not composed of letters perfectly formed or aligned with one another. This negative relationship between handwriting speed and handwriting quality was also observed in both cohorts, with significant correlations at different grades in the single-word dictation task (coh1: Grades 2 and 4; coh2: Grades 3 and 5). This relationship is in line with a commentary published by Graham (2018), raising the dilemma that students face between writing fast or writing neatly. While this observation was made at the text level for young adults, the present study addressed for the first time the issue of the relationship between speed and handwriting quality in children at the word level.

In the younger cohort (Grades 2–4), the results revealed that children's handwriting speed at the beginning of the study was negatively related to the development of the same ability. In other words, the improvement in speed execution between Grade 2 and Grade 4 was greater for children who started with slow handwriting. This is not surprising, since slow children had greater room for progress than children who had more advanced and automated handwriting abilities at the beginning of the study.

Our analyses did not bring any significant result regarding the developing relationship between spelling and handwriting quality. However, a significant cross-sectional positive correlation between spelling accuracy and handwriting quality, limited to the beginning of the study, was found in the younger cohort only. Significant correlations were present between spelling at the standardized task (i.e., sentence dictation) and quality in the standardized task (i.e., text copying) but not in the single-word dictation context. This relation disappeared after Grade 3, suggesting that spelling accuracy and handwriting quality are no longer related once children have more automatised handwriting, which can be understood in the framework of McCutchen's capacity of writing theory (1996). This interpretation is congruent with the above-mentioned findings demonstrating that graphic complexity negatively impacted spelling accuracy only in Grade 2 (coh1) and not in Grade 3 (coh2).

## Study Limitations and Perspectives

This section addresses the present study's limitations, which can be useful for researchers designing experiments in the field of writing development. The first limitation concerns the experimental task used in the present experiment, which was a single-word dictation task. Word dictations are exclusively related to the context of spelling evaluation at school, which limits the ecological validity of such writing tasks. Adding data from a sentence dictation task could be a way of assessing children's writing abilities in a more ecological way, since writing in most school activities (e.g., text generation, taking notes during lessons) and personal contexts (e.g., writing letters) is not limited to single-word writing. Therefore, we strongly encourage researchers to collect longitudinal data on spelling, handwriting speed, and handwriting quality in various writing contexts.

A second limitation of the present study concerns the measurement of speed, which reflects the speed of graphomotor execution. Future experiments should consider several dynamics of handwriting to investigate not only the graphomotor speed of execution but also the total writing durations and pause durations. Having measures that better represent the time course of writing will help in understanding how spelling and handwriting are related, since latency times and pauses within writing are typical manifestations of orthographic processes (Sumner et al., 2013).

A third limitation is the relatively small window of time covered by the study. Each cohort was followed for a period of 3 years, from Grade 2 to Grade 4 in coh1 and from grade 3 to Grade 5 in coh2. Past literature has concluded that handwriting legibility development may be limited to the very beginning of explicit teaching. It would have been relevant to start the study in Grade 1 to capture the critical improvement phase of handwriting quality. Along the same lines, since speed of handwriting and spelling accuracy continue developing even in the later grades, it would have been interesting to keep following the same participants at least until these skills reach a plateau. The current study did not enable us to understand when each transcription skill reaches a mature level. However, designing longitudinal experiments covering such a large window of time can seem unrealistic because longitudinal designs are known to be challenging to conduct. Nevertheless, even if they cannot cover the whole period of childhood development, more longitudinal studies are needed to better understand writing development.

A fourth limitation concerns the limited assessment of teaching and motivational factors. Indeed, even if children all came from two primary-schools in which teachers were using the same educational method for spelling and handwriting instruction, it would have been interesting to gather more information about teaching practices. In the same vein, it would have been useful to collect data regarding participants' motivation toward writing. These factors, i.e., teaching effect and motivational variables (Camacho et al., 2021), could also explain part of writing development, as well as the significant differences highlighted between cohorts.

Finally, readers should keep in mind that the present longitudinal study involved data collected in a cursive handwriting context in a French-speaking sample, and French orthography is opaque. Handwriting cursive and script styles are known to differ in terms of graphomotor gestures, i.e., continuity within the word vs. pauses between each letter. Moreover, differences in the pace of spelling have already been documented in accordance with language consistency. Therefore, the relationship between spelling and handwriting could possibly develop differently in script and cursive contexts and in opaque compared to transparent orthographies. By replicating the study in other educational and linguistic contexts, future research could highlight how the development of spelling and handwriting is dependent on language consistency as well as handwriting style.

## Educational Implications

The present study highlights the long-lasting challenge represented by writing development. The two transcription skills, word spelling and handwriting, were still growing until the end of the study, i.e., Grades 4 and 5. While handwriting is taught only in the very beginning of primary school (Graham et al., 2008), the results support keeping handwriting practice at the center of focus longer. Past experiments have already highlighted the need to focus more attention on the development of graphomotor skills during primary school (Feder and Majnemer, 2007; Graham, 2010), but teachers frequently report that they lack tools and methods to teach handwriting skills (Graham et al., 2008). By highlighting the influence of graphic complexity on writing outcomes, the present findings encourage future experiments to assess the efficacy of teaching handwriting with a particular focus on graphically complex segments. An important finding arising from the present study concerns the reverse relationship between handwriting speed and quality. This finding suggests that, at the single word level, having fast handwriting is associated with poorer handwriting quality. In contrast, children with high handwriting quality write slowly. To understand this finding, one can consider his/her own adult handwriting: to be efficient when taking notes, speed is inevitably favored over handwriting quality.

Overall, this longitudinal study highlighted different facets of the long-lasting challenge implied by learning to write words. While spelling and handwriting speed are positively related, handwriting speed and handwriting quality are negatively related during development. Through the study of orthographic and graphic complexity, the results showed that spelling, handwriting speed, and handwriting quality influence each other during writing. Notably, graphic complexity had a significant impact on the development of handwriting speed and on spelling accuracy in beginning writers (Grade 2). Improvement in one ability may positively influence the other by reducing cognitive constraints. Therefore, handwriting automatisation, by reducing the cognitive load for children, appears crucial for writing development. However, even with more advanced handwriting skills, it is important to keep in mind that each writing production is the result of a multitude of cognitive processes active in parallel and influencing each other. Both transcription skills, i.e., spelling and handwriting, need time and practice opportunities to develop. Even if spelling and handwriting progress greatly during primary school, this study suggests that mature levels are not yet reached at the end of primary school.

## Data Availability Statement

The datasets generated for this study are available on request to the corresponding author.

## Ethics Statement

The studies involving human participants were reviewed and approved by Ethical Committee of the Psychological Sciences Research Institute. Written informed consent to participate in this study was provided by the participants' legal guardian/next of kin.

## Author Contributions

CG developed the research questions, designed the experiment, performed the experiment, scored the tasks, interpreted the results, drafted, and revised the manuscript. MP analyzed the data, discussed data interpretations, wrote the results section, and revised the manuscript. MVR developed the research questions, designed the experiment, discussed data interpretations, and revised the manuscript. All authors contributed to the article and approved the submitted version.

## Conflict of Interest

The authors declare that the research was conducted in the absence of any commercial or financial relationships that could be construed as a potential conflict of interest.

## Publisher's Note

All claims expressed in this article are solely those of the authors and do not necessarily represent those of their affiliated organizations, or those of the publisher, the editors and the reviewers. Any product that may be evaluated in this article, or claim that may be made by its manufacturer, is not guaranteed or endorsed by the publisher.
