# Supplementary material for: How Do Spelling, Handwriting Speed, and Handwriting Quality Develop During Primary School? Cross-Classified Growth Curve Analysis of Children's Writing Development
Source: Front Psychol. 2021 Jul 23;12:685681. doi: 10.3389/fpsyg.2021.685681 (PMC8343101; doi:10.3389/fpsyg.2021.685681)
Supplement: Supplementary file 1 [file Table_1.docx]

Appendix A. Average Performance at the Word-Dictation Task per Cohort according to Orthographic complexity: Orthographically Simple Words and Orthographically Difficult Words

|  |  | Cohort 1 | |  | Cohort 2 | |
| --- | --- | --- | --- | --- | --- | --- |
|  |  | O simple | O difficult |  | O simple | O difficult |
| Spelling Accuracy (success rate) | | | | | |  |
| T1 |  | 71.8% | 3.2% |  | 88.5% | 18.0% |
| T2 |  | 86.3% | 14.2% |  | 93.5% | 32.3% |
| T3 |  | 88.9% | 30.6% |  | 94.1% | 48.0% |
| Handwriting Speed (cm/s) | | | |  |  |  |
| T1 |  | 1.36 (0.52) | 1.32 (0.55) |  | 1.70 (0.63) | 1.56 (0.66) |
| T2 |  | 1.78 (0.61) | 1.63 (0.63) |  | 1.74 (0.62) | 1.66 (0.63) |
| T3 |  | 2.15 (0.79) | 1.99 (0.76) |  | 1.99 (0.68) | 1.90 (0.73) |
| Handwriting Quality (number of graphic errors) | | | | | |  |
| T1 |  | 1.79 (0.97) | 1.61 (0.95) |  | 1.57 (0.88) | 1.44 (0.84) |
| T2 |  | 1.58 (1.12) | 1.37 (1.01) |  | 1.28 (1.06) | 1.16 (0.96) |
| T3 |  | 1.97 (1.11) | 1.76 (1.07) |  | 1.63 (0.98) | 1.46 (0.85) |

*Note.* Cohort 1: T1 = Grade 2; T2 = Grade 3; T3 = Grade 4, Cohort 2: T1 = Grade 3; T2 = Grade 4; T3 = Grade 5

Appendix B. Average Performance at the Word-Dictation Task per Cohort according to Graphic Complexity: Graphically Simple Words and Graphically Difficult Words

|  |  | Cohort 1 | |  | Cohort 2 | |
| --- | --- | --- | --- | --- | --- | --- |
|  |  | G simple | G difficult |  | G simple | G difficult |
| Spelling Accuracy (success rate) | | | |  |  |  |
| T1 |  | 44.4% | 30.6% |  | 57.3% | 49.2% |
| T2 |  | 55.5% | 45.0% |  | 65.4% | 60.4% |
| T3 |  | 63.6% | 56.0% |  | 73.6% | 68.6% |
| Handwriting Speed (cm/s) | | | |  |  |  |
| T1 |  | 1.34 (0.55) | 1.34 (0.51) |  | 1.73 (0.69) | 1.52 (0.58) |
| T2 |  | 1.81 (0.65) | 1.59 (0.57) |  | 1.75 (0.66) | 1.65 (0.59) |
| T3 |  | 2.18 (0.80) | 1.96 (0.74) |  | 2.04 (0.73) | 1.85 (0.67) |
| Handwriting Quality (number of graphic errors) | | | | |  |  |
| T1 |  | 1.52 (0.89) | 1.89 (1.00) |  | 1.36 (0.80) | 1.65 (0.89) |
| T2 |  | 1.27 (0.99) | 1.68 (1.11) |  | 1.04 (0.93) | 1.40 (1.06) |
| T3 |  | 1.65 (1.02) | 2.08 (1.12) |  | 1.36 (0.84) | 1.73 (0.96) |

*Note.* Cohort 1: T1 = Grade 2; T2 = Grade 3; T3 = Grade 4, Cohort 2: T1 = Grade 3; T2 = Grade 4; T3 = Grade 5
